# Current and Future Approaches to Mitigate Conflict between Humans and Asian Elephants: The Potential Use of Aversive Geofencing Devices

**DOI:** 10.3390/ani12212965

**Published:** 2022-10-28

**Authors:** Surendranie Judith Cabral de Mel, Saman Seneweera, Ruvinda Kasun de Mel, Ashoka Dangolla, Devaka Keerthi Weerakoon, Tek Maraseni, Benjamin Lee Allen

**Affiliations:** 1 Institute for Life Sciences and the Environment, University of Southern Queensland, Toowoomba, QLD 4350, Australia; 2 National Institute of Fundamental Studies, Kandy 20000, Sri Lanka; 3 Faculty of Veterinary and Agricultural Sciences, The University of Melbourne, Parkville, VIC 3010, Australia; 4 Centre for Behavioural and Physiological Ecology, Zoology, University of New England, Armidale, NSW 2351, Australia; 5 Department of Veterinary Clinical Sciences, University of Peradeniya, Peradeniya 20400, Sri Lanka; 6 Department of Zoology and Environmental Sciences, University of Colombo, Colombo 00300, Sri Lanka; 7 Northwest Institute of Eco-Environment and Resources, Chinese Academy of Sciences, Lanzhou 730000, China; 8 Centre for African Conservation Ecology, Nelson Mandela University, Port Elizabeth 6034, South Africa

**Keywords:** aversive conditioning, *Elephas maximus*, human-wildlife conflict, virtual fencing, wildlife management

## Abstract

**Simple Summary:**

Conflict between humans and Asian elephants is a major conservation issue. Here we discuss common tools used to manage human-elephant conflict (HEC) in Asia and the potential of animal-borne satellite-linked shock collars or Aversive Geofencing Devices (AGDs) for managing problem elephants. Most current HEC mitigation tools lack the ability to be modified to accommodate needs of elephants and therefore are sometimes unsuccessful. AGDs currently used to manage livestock movement can be adapted to mitigate HEC to overcome this problem. AGDs can constantly monitor animal movements and be programmed to deliver sound warnings followed by electric shock whenever animals attempt to move across virtual boundaries demarcated by managers. Elephants fitted with AGDs are expected to learn to avoid the electric shock by associating it with the warning sound and move away from specified areas. Based on the potential shown by studies conducted using AGDs on other wild species, we suggest that experiments should be conducted with captive elephants to determine the efficacy and welfare impact of AGDs on elephants. Further, assessing public opinion on using AGDs on elephants will also be important. If elephants can learn to avoid virtual boundaries set by AGDs, it could help to significantly reduce HEC incidents.

**Abstract:**

Asian elephants are a principal cause of human-wildlife conflict. This results in the death/injury of elephants and humans and large-scale crop and property damage. Most current human-elephant conflict (HEC) mitigation tools lack the flexibility to accommodate the ecological needs of elephants and are ineffective at reducing HEC in the long-term. Here we review common HEC mitigation tools used in Asia and the potential of Aversive Geofencing Devices (AGDs) to manage problem elephants. AGDs can be configured to monitor animal movements in real-time and deliver auditory warnings followed by electric stimuli whenever animals attempt to move across user-specified virtual boundaries. Thus, AGDs are expected to condition elephants to avoid receiving shocks and keep them away from virtually fenced areas, while providing alternative routes that can be modified if required. Studies conducted using AGDs with other species provide an overview of their potential in conditioning wild animals. We recommend that the efficacy and welfare impact of AGDs be evaluated using captive elephants along with public perception of using AGDs on elephants as a means of addressing the inherent deficiencies of common HEC mitigation tools. If elephants could be successfully conditioned to avoid virtual fences, then AGDs could resolve many HEC incidents throughout Asia.

## 1. Introduction

Asian elephants *Elephas maximus* (Linnaeus 1758) once inhabited areas between the Euphrates and Tigris Rivers in west Asia to the Yangtze-Kiang River in China [1], but now inhabit a much smaller range within 13 countries: Bangladesh, Bhutan, Cambodia, China, India, Indonesia, Laos, Myanmar, Nepal, Sri Lanka, Thailand and Vietnam [2]. The total global population is estimated to be about 48,323 to 51,680 individuals, of which almost 75% of the population is found in India and Sri Lanka [3]. There is also a captive Asian elephant population of approximately 14,930 to 15,130 in range countries [3] and another ~1000 maintained in zoos outside range countries [4]. Asian elephants (hereafter elephants) are worshiped as a god in Hinduism and have an important role in Buddhism, two of the main religions in the region [5,6,7]. Ancient kings maintained thousands of elephants as work animals and warriors; they also traded and gifted them between countries [1,6,8,9,10]. In contemporary societies, captive elephants are commonly kept in temples and are used in ceremonial and religious rituals; they are also used in the logging and tourism industries [11,12,13]. Thus, elephants have played an important role in Asian cultural heritage since ancient times.

Despite the elephant conservation legislation imposed, various anthropogenic activities have continued to threaten the survival of elephants. Legal and illegal capture and illicit trade of elephants to supplement captive populations occur in several nations, which contributes to the decline of elephant numbers in the wild [14,15,16]. Hunting elephants for ivory, meat, hair, tail, bones and skin further poses a major threat [17,18,19]. Thus, elephants are listed in Appendix 1 of the Convention on the International Trade in Endangered Species (CITES), prohibiting international trade of elephants and elephant parts. Elephants are also listed as Endangered on the International Union for Conservation of Nature (IUCN) Red List of Threatened species [20] given elephant distribution has fragmented and declined considerably over the past few decades [21,22,23,24,25]. Many Asian countries with extant elephant populations also have high human population densities and developing economies [26]. These countries focus on large-scale and rapid industrial development and expansion projects which inevitably convert areas of wilderness to permanent human settlements, commercial zones and agricultural lands [27,28,29,30,31]. The resulting fragmented and heterogenous landscapes thus increases the frequency of interactions between humans and elephants [32,33], which is the root cause of human-elephant conflict (HEC).

Many elephant and human lives are lost as a consequence of HEC with highest numbers recorded in India and Sri Lanka, where an average of 124 elephants and 571 humans in India [34] and 263 elephants and 81 humans in Sri Lanka [35] are killed annually. HEC related elephant deaths result from gunshot injuries, poisoning, electrocution from illegal electric fences, accidents such as falling into agricultural wells or abandoned gem pits, and collision with trains [29,36,37]. Exposure to human disturbances increases stress levels of elephants which effects their reproductive success [38]. Many infant elephants are orphaned as a result of HEC as well [29]. Injury and death of humans often occur during chance encounters, particularly at night when humans confront and seek to deter crop-raiding elephants and those that damage houses to feed on stored grains [39,40], when people step out at dawn for toileting [41], enter forests to extract resources [42], or due to irresponsible behaviour [35]. Crop raiding is the main source of conflict between humans and elephants [6,43] as elephants raid many different cultivated crops such as rice, corn, millet, maize, sugar cane, vegetables, fruits and even coconut palms [40,44,45,46,47]. Affected people experience substantial economic losses and governments spend large sums of money in compensation payments for elephant impacts [48,49,50]. Apart from loss of lives, crops and property, there are also social and psychological effects which are often not accounted for when assessing HEC impacts [51,52]. Thus, mitigating HEC remains a key challenge for many of the elephant range countries.

Various tools and strategies are used to mitigate HEC and keep damage-causing elephants away from crops and other human-dominated areas [39,53]. The occurrence and frequency of HEC has increased despite mitigation efforts by governments and conservation organisations [35,54,55] due to various weaknesses in the HEC mitigation methods presently used. Current methods are mainly focussed on managing the symptoms of the conflict, but successful mitigation of HEC requires a greater focus on the root causes [56]. Elephants occupy large home ranges and travel long distances, depending on resource availability and reproductive status [32,57,58]. For example, elephants have larger home ranges in fragmented landscapes compared to non-fragmented habitats as elephants travel more in search of food and water due to their limited availability [32]. Further, during the musth period, male elephants cover much wider ranges in search of mates compared to the non-musth period [57]. Therefore, maintaining habitat connectivity is vital for HEC mitigation and elephant conservation [59]. For this, understanding and accommodating human and elephant behaviour to prevent HEC from occurring is extremely important [60]. Developing innovative tools and strategies that can reliably keep problem-causing elephants away from humans and crops, are dynamic and flexible enough to be modified according to elephant and human needs, and pose minimum welfare impacts to elephants are sorely needed.

Satellite-linked electric shock collars or Aversive Geofencing Devices (AGDs) can automatically deliver a warning sound followed by an electric shock as an animal reaches a virtual boundary, and have been successfully used in managing livestock movement [61,62,63]. The earliest reference of using AGDs on a wild species is for coyotes *Canis latrans*, in 1976 where three out of the four shock-collared animals learnt to avoid black domestic rabbits and prey on white rabbits after 3–5 shocks [64]. AGDs appear to have the potential as an HEC mitigation tool where wildlife authorities could fit them on identified “problem” elephants [65], and create and modify virtual fences based on human and elephants’ needs. If virtual fences can be created appropriately for high HEC areas and problem elephants can successfully learn to avoid them, then AGDs may become a very powerful HEC mitigation tool.

Here we briefly review the use of common approaches to manage conflict between humans and elephants across Asia, highlighting their function and drawbacks. We then discuss the potential use of AGDs as a means to address these drawbacks and sustainably mitigate HEC. We further describe important research needs that require addressing to advance the use of AGDs on elephants. Our aim is to highlight the similarities and differences between AGDs and other HEC mitigation tools and outline a pathway forward for the trial and development of AGDs on elephants.

## 2. HEC Mitigation Tools

A wide array of tools are used in Asia to mitigate HEC and several reviews have been published in the recent past on various aspects of HEC mitigation [56,59,60,66,67]. These have highlighted some progress, but have also highlighted a series of weaknesses in current approaches, which we discuss under five categories: (1) exclusion (2) removal of problem elephants, (3) early warning systems, (4) human centric methods and (5) habitat management, summarised in Table 1.

Exclusion of elephants from conflict areas or restricting elephants to protected areas aims to keep elephants away from humans and their interests and is ostensibly intended to avoid the need for the direct killing of elephants in accordance with cultural and societal expectations. Exclusion is often achieved by aversive conditioning where animals learn to associate a particular behaviour with an unpleasant stimuli, and hence cease or modify that behaviour [135,136,137]. A multitude of aversive stimuli are used against elephants which they learn to avoid by associating it with a warning stimulus (Table 2). However, large elephant populations live outside protected areas and boundaries created by humans do not always align with the ecological boundaries that elephants adhere to [22,118,138,139]. Thus, excluding animals from human habitats will not successfully mitigate HEC unless alternative routes and habitats are provided.

As an alternative to excluding elephants from human habitats, identified problem elephants may be physically removed from a population by either killing or translocating them. Large-scale culling of elephants is no longer sanctioned in Asian elephant range countries [75], but massive culling and translocation operations conducted in Africa revealed long term social disruption in the remaining younger elephants who experienced the traumatic event [140]. One reason for large-scale culling of elephants in Africa is to manage large elephant populations that have exceeded carrying capacities [133] because it would otherwise cause irreversible damage to vegetation due to overutilisation by elephants, affecting the food availability for other species [141]. However, such vegetation transformation has not been observed by Asian elephants [6]. Removal of elephants may negatively affect the stability of the source population [78] and removed elephants may be replaced by other elephants which continue the conflict [18]. Translocation of elephants may be recommended as a last resort to save individuals or very small groups isolated from other elephant populations [78]. The removal of elephants by either killing or translocation also addresses only the symptom of HEC and is typically considered unfeasible and ethically unacceptable.

Various types of early warning systems are sometimes implemented to mitigate HEC, ranging from vigilance by farmers occupying traditional watchtowers to monitoring elephants using various remote sensing technologies (Table 1). The use of more modern and emerging technologies, are gaining a lot of interest and if financial and technological barriers can be overcome, they would immensely help in avoiding encounters with elephants [118]. However, early warning systems would still require humans to respond and chase the elephants away unless they are coupled with an aversive stimulus of some kind. A better tool would be an early warning system that would automate an effective aversive response without any human interaction with elephants.

Human centric methods are focused on encouraging human-elephant co-existence and developing tolerance towards elephants by providing financial relief or by educating stakeholders. The knowledge gap about HEC and the endangered status of elephants may intensify the conflict [142]. Even though financial relief has an immediate effect and addresses only the symptom of the problem, along with creating awareness, it helps to gain continuous support of stakeholders to mitigate HEC both in the short and the long term.

Habitat management through managing ecological corridors and enriching protected areas expects to reduce human-elephant interactions by reducing the need for elephants to venture into human-dominated habitats. Elephants are forest animals, but edge species, preferring habitats with intermediate disturbance rather than undisturbed forests [143,144,145,146]. Elephants are often attracted to landscapes disturbed by humans, thereby increasing the chances of HEC [31]. Alternatively, elephants may enter human-dominated landscapes simply because it is a connecting path leading to other resources such as water and mates [60]. Therefore, giving priority to proper land use planning and improving connectivity between elephant habitats [59] will be more effective to assist dispersal of elephants with minimum human encounters.

Overall, many of the current mitigation efforts either address the symptoms of HEC or are not dynamic or flexible enough to be modified as needs change, and therefore are successful only in the short term or are not sustainable [66]. Based on the functions and drawbacks highlighted above, the following can be suggested as ideal characteristics or objectives of tools that could successfully mitigate HEC:

1. Prevents HEC incidents before they occur
2. Keeps elephants in or out of designated areas
3. Targets specific individuals or small family groups
4. Does not require the death of the animal
5. Produces minimal harm to elephants
6. Does not harm or impede non-target species
7. Does not require the construction of permanent or immovable structures
8. Can be altered, moved, or removed as needed
9. Is long-lasting or sustainable
10. Is automated, or does not require substantial human input
11. Is inexpensive or cost-effective
12. Is culturally and socially acceptable

With current mitigation tools each having only some of these characteristics (Table 1), developing new and innovative tools remains a key priority for management and research. AGDs are one such potential tool and are essentially a combination of an exclusion method using aversive conditioning stimuli and an early warning system where people can be notified when elephants are nearby, addressing many of the above characteristics. AGDs have previously been suggested as a potential HEC mitigation method [108], but little progress has been made since that time.

## 3. Animal-Borne Aversive Geofencing Devices (AGDs): A Potential Tool for Reducing Conflict with Asian Elephants?

### 3.1. Use of AGDs on Domestic Animals

AGDs have been used on domestic pets (i.e., dogs) and livestock for many decades [147]. The first commercial AGD was patented in 1973 for dogs, where a hidden, signal-emitting wire placed around a predetermined boundary triggered the animal-borne collar to deliver an electric shock when the animal approached the wire [148]. In this way, dogs were contained in a residential backyard without the need for a visible fence. These dog training collars were modified and first used on livestock in 1987 when goats (*Capra hircus*) were also successfully contained in a designated area without a visible fence [149]. Since then, AGDs that are manually controlled or ones that use proximity based sensors have been used on other livestock species like cattle *Bos taurus* [150,151] and sheep *Ovis aries* [152,153] as well. Although generally considered effective, this approach still reflected the logistical limitations of a physical electric fence, including an inability to modify virtually fenced areas easily and establishing virtual fences in large landscapes.

Technical development of AGDs has evolved since then and modern AGDs are now able to deliver stimuli automatically in conjunction with real-time GPS tracking, user alerts, and data logging capabilities similar to most standard GPS tracking devices. They have proven to successfully restrict livestock movement to large and dynamic user-specific areas without proximity-based sensors [154], overcoming the limitations of earlier attempts. Farmers can now define a virtually fenced area, upload these boundaries onto an animal-borne device, deploy it on an animal, and then remotely monitor and control the movement of that animal in real-time. Animals attempting to cross a virtual boundary are first given an audible warning, which escalates if ignored, and then the ignored warnings are followed with an electric shock if the virtual fence is breached, shepherding the animal back to the safe zone if needed [62,63,155]. The locations of such virtual fences are temporally and spatially flexible, and therefore allow managers to change or alter the location of safe zones as needed. In other words, users can remotely move their animals from one location to another or allow/deny animal access to one location or another without being present. Experiments have shown that cattle and sheep learn to associate electric shock with the warning sound emitted by the collar after just a few attempts [153,156]. Key findings of some research conducted on virtual fencing with AGDs on livestock published from 2017–2022 (~last 5 years) are given in Appendix A (Table A1).

### 3.2. Use of AGDs on Wildlife

Even though responses of elephants to AGDs may vary from that of other animals, reviewing what is known from studies on other wild species may provide some insight into the potential and challenges that could be expected in conditioning elephants using AGDs. Scientific material published in the past 30 years (between 1993–2022) in the English language related to the use of AGDs on wild species were searched in Web of Science and Google Scholar using the following search string: (“shock collar*” OR “electric collar*” OR “training collar*” OR “electronic collar*” OR “e-collar*” OR “automated collar*” OR “virtual fencing collar*”) AND (“wildlife management” OR “wildlife conservation” OR “*wildlife conflict*” OR “predator management” OR “crop damage”). The initial search (last performed on 16th August 2022) resulted in 127 records. The titles and abstracts of each document was screened and eight empirical studies that involved direct experimentation with animal-borne electric shock collars on a wild species were extracted. References within articles were checked, and four articles missing from the initial list were added. The resulting list of articles (n = 12) are summarised in Table 3.

According to the search results, research using AGDs has been conducted with five wild species: coyotes, grey wolves *Canis lupus*, dingoes *Canis familiaris*, island foxes *Urocyon littoralis* and black-tailed deer *Odocoileus hemionus*. The total number of wild animal studies conducted over the past 30 years are very few compared to the large number of studies available on livestock (see Appendix A). Even though most studies (n = 9) used an automatic shock delivery method, they all used proximity-based sensors, limiting the area of shock collars’ use. While three studies showed longer-term effectiveness of shock collars in conditioning animals after collars were deactivated [157,160,164], three other studies showed that animals returned to showing their undesirable behaviour sometime after the deactivation of shock collars [161,163,165]. Only two studies [163,165] used sound as a warning stimulus before delivering a shock, and both these studies showed that it is possible to condition animals to avoid shock using a sound warning. Effectiveness and battery life of shock collars may also be augmented by coupling a warning (lights or sound) before electric shock is delivered [163]. These studies also emphasized that use of AGDs is a better alternative than lethal control.

Many drawbacks and limitations were highlighted in these studies such as skin necrosis due to electrodes, irritation due to the collar belt material [157,159,166,167], improper fitting of collars or displacement of electrodes [161], limited battery life [157,161,162], the need for automatic activation of the collar [157], limited range of shock collar activation [159], inconsistency in shocking devices [161], and the need to reduce the weight of the shock unit [162]. Logistical difficulties of working with wild animals also affected the success of studies [168]. Further, extensive effort and high cost of collaring wild animals [159,163] could limit the number of animals that can be targeted using this approach. Variability in responses to stimuli by individual animals [168] that may also have occurred due to inconsistent shock delivery [161] was emphasized. These studies were also affected by low sample sizes and low number of trials, limiting the opportunity to test the devices properly or condition the animals [167], resulting in inconclusive outcomes. The sample size in most studies was less than 10 individuals with only a few exceptions [159,162]. Automatically activated AGDs that can be deployed over large heterogenous landscapes have not yet been tested with wild species. Investigating and overcoming these drawbacks will be essential before AGDs can be reliably implemented as an HEC mitigation tool.

### 3.3. AGDs as a Potential HEC Mitigation Tool

AGDs could help prevent HEC incidents before they arise if elephants learn to recognise the warning stimuli and predict the receipt of the electric shock and avoid it by moving away. This will minimise direct human interaction with elephants and prevent HEC incidents. AGDs may therefore be a good alternative when it is impractical to permanently erect electric fences in large areas [159] given their application does not require development of permanent structures, allowing wildlife managers to easily create, move, modify, and remove the virtual fences when needed. Elephants are highly intelligent and have superior cognitive abilities [169,170], making them ideal candidates for aversive conditioning with AGDs.

**Figure 1:**
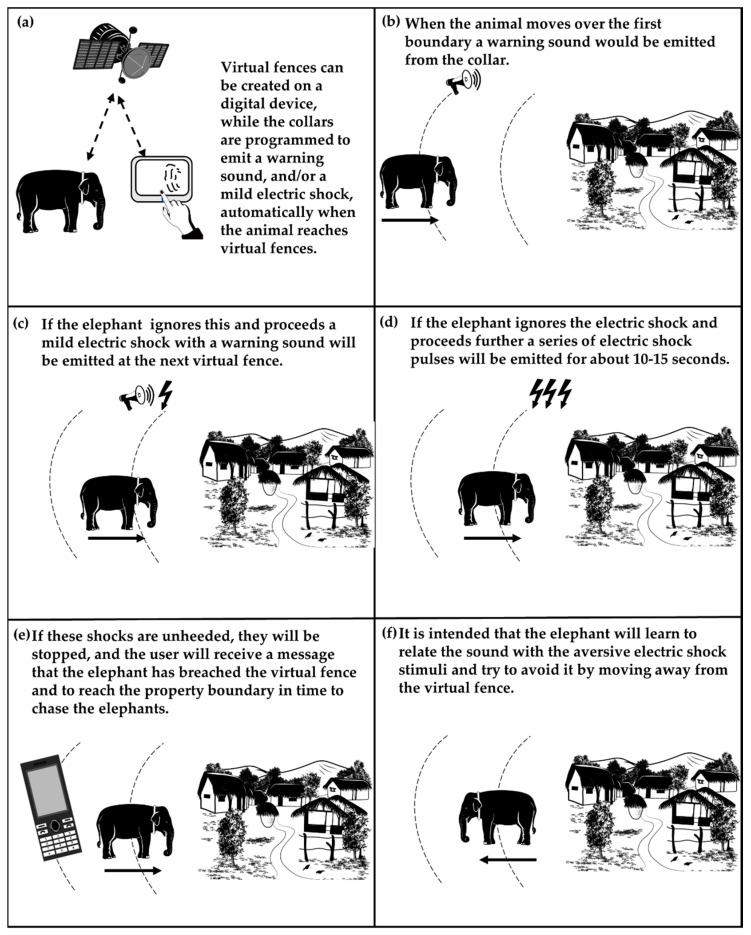
Conceptual diagram of how Aversive Geofencing Devices (AGDs) are expected to work to manage movement of a wild elephant. (**a**) Virtual fences are drawn on a digital device. (**b**) Sound warning is delivered as the elephant fitted with an AGD approaches first virtual fence. (**c**) Elephant approaches second virtual fence and receives both sound and electric stimuli. (**d**) Elephant proceeds further and receives electric shock as pulses. (**e**) A warning message is sent to villagers’ mobile phones if the elephant ignores the electric shocks and proceeds further. (**f**) Elephant learns to turn away and avoid receiving electric shocks after few instances.

While the concept of testing AGDs on wild elephants to manage their movement may be attractive, elephants may not respond to the electric stimuli the same way livestock do and information available on other wild species may not be sufficient to foresee the potential of AGDs as an HEC mitigation tool. Virtual fences will also have to be established in much larger, heterogenous and complex landscapes than those that livestock are typically managed in. Figure 1 shows a conceptual illustration of how AGDs are expected to work to mitigate HEC. Conditioning elephants using AGDs is a complex process. Electric shocks are received by the elephant in the first few instances, and the probability of the unwanted behaviour (e.g., moving towards a village) is expected to decrease in the future as the animal learns to avoid the electric shock [171]. However, if the unwanted behaviour would be fully extinguished and whether elephants would move in the desired direction in the absence of a visual stimulus or a physical barrier is unknown. Unlike other wild species tested so far, an agitated elephant moving towards a village or agricultural land could create an unpredictable and potentially dangerous situation. AGDs should have a sense of directionality which is achieved by applying the stimuli only when animals move towards the exclusion zone rather than their location per se, so that they can learn the virtual fences accurately [156]. This will allow the animal to predict and control the receipt of the aversive stimuli while minimising the stress [172,173] and move in the desired direction. Planning, designing and monitoring virtual fences should also be done carefully. Baseline studies of land use and movement of both humans and elephants needs to be evaluated on a case-by-case basis [60] and all stake holders such as authorities, researchers and villagers should work together in planning and designing the location of virtual fences. These virtual fences should then be continuously monitored and evaluated and be modified as and when appropriate. Keeping elephants in or out of a designated area using AGDs would be possible by designing virtual fences in such a way that a safe ‘escape route’ is clear and available.

Fitting AGDs on wild elephants would also be a complex and costly process [119], so AGDs cannot be deployed on all elephants. Since most crop raiding elephants are lone males [43,174,175], installing AGDs on identified problem-causing lone elephants and matriarchs of herds would be more appropriate. Social facilitation could be expected to occur in group living, long lived animals like elephants where a matriarch collared with an AGD may lead the rest of the herd to avoid the electric shock associated with the virtual fence [147,176]. Learning to avoid virtual fences through social facilitation has been shown to occur in cattle and sheep with only a proportion of the animal group collared with AGDs [177,178]. The potential for wolves to learn through social facilitation was also shown where the rest of the pack members not wearing shock collars learnt to avoid a baited site [164]. Monitoring elephant movement and habitat use using GPS collars is conducted widely in Asian elephant range countries [17,31,179,180]. Given that AGDs also fulfil the same function of a GPS collar, fitting AGDs may be conducted at a similar scale as part of ongoing research that involves GPS collaring on selected elephants.

## 4. Progressing the Development of AGDs as a HEC Mitigation Tool

### 4.1. Developing and Testing the Efficacy of AGDs on Elephants

Elephants appear to be good candidates for the use of AGDs, but elephant’s large size, strength, speed, and potentially dangerous behaviour poses a risk in testing AGDs on elephants. Individual variability in their capacity for learning and response to the electrical stimuli might also be expected [63,152,153,168,181]. Furthermore, elephants have several different sensitive locations on the neck where electric probes may be more or less helpful in influencing animal movement or be avoided to prevent any harm to the elephant [182]. How individuals perceive the pain from the electric shock [183] and their temperament [184] may also vary. Hence, there is no guarantee that use of AGDs will be immediately successful for elephants. To determine the efficacy of AGDs on elephants, pilot studies should be conducted using captive elephants under controlled conditions [158,185,186]. Identifying the most suitable location on the neck to deliver the shock, and the safest appropriate strength of the shock, should be of primary research interest [153]. Field trials will then need to be conducted to understand the learning ability of elephants to associate the warning signals with the electric shock and avoid it. Negative reinforcement is often practiced by mahouts during training and handling of captive elephants in Asia [187,188,189]. However, safety of the mahout, relationship between mahouts and elephants and mahouts’ perception on testing AGDs on captive elephants should be considered during field trials. Exploration of the potential for captive elephants to learn through social facilitation would also be beneficial prior to testing of AGDs on wild elephants. Responses by captive elephants may not entirely represent wild elephant responses, but preliminary investigations with captive animals would still help resolve several uncertainties prior to work on wild elephants.

The longevity of AGDs must be considered given that frequent replacement of collars on wild elephants is not possible. GPS collars have limited battery life and are typically scheduled to collect GPS points every few hours [119]. However, AGDs will require real-time positioning of elephants and also generate sound and electric shock, thereby consuming a lot of battery capacity. Exploring options of harvesting energy using solar power, motion and body heat may be advantageous [147,190,191]. Maintaining uninterrupted communication between satellites and AGDs despite topographic barriers should be investigated [62], and the durability of the AGD is also an important factor requiring attention. In addition to being waterproof, the device may also have to be resistant to mud. AGDs should also be able to withstand strong movements such as head shaking or collar shaking using the trunk or rubbing of the collar against hard surfaces. Each of these issues need further exploration before AGDs will be ready for operational deployment on wild elephants.

### 4.2. AGDs and Elephant Welfare

AGDs typically expose animals to a high voltage electric shock with a very low amperage, delivered as pulses for a few milliseconds at a time [157,183], thereby minimising harm to the animal [192]. The strength of the shock from AGDs would also be much lower than what is received from electric fences [193]. Electricity will pass through and pain will be felt only between the contact points of the electrodes [194]. Further, when using AGDs the aversive stimulus is felt by the fewest number of possible animals and does not affect non-target individuals or species. Using devices that intentionally expose animals to pain naturally raise concerns about the ethical and welfare implications for the animal [158,195,196]. It might be expected that animals would show acute stress responses during early stages of learning, but after learning has occurred and animals know how to avoid the stimuli effectively, chronic stress levels should be no different from normal baseline levels [186,197]. Several studies have explored physiological stress levels using cortisol hormone and behavioural responses to understand the welfare of animals in relation to aversive conditioning [154,172,173,193,198,199,200]. If animals continue to show chronic stress responses and inability to learn, the experiment may need to be modified or discontinued with those animals [197]. Measuring cortisol hormone and behavioural time budgets are commonly used to assess stress levels of elephants [201,202,203]. Therefore, during preliminary studies, similar analysis should be done, as an indicator of welfare impacts associated with AGDs on elephants.

### 4.3. Public Acceptance for Using AGDs on Elephants

Obtaining acceptance of all stakeholders, local communities, line agencies, local administration and government is required to mainstream the use of AGDs. All approaches to managing HEC cause some sort of pain, distress, or disruption to elephants, but public acceptance of AGDs depends on how these welfare impacts compare to or are perceived to be compared to other HEC mitigation tools (Table 1). Use of electronic training collars on animals is not a common practice in Asian elephant range countries. Therefore, public reluctance to accept a novel technology may also be a challenge. In addition to the efficacy and welfare, successful adoption of new mitigation tools will be contingent on the probability of people to perceive it favourably, the capacity for the relevant stakeholders to implement or maintain it, and their ability to expand and adapt it on a wider scale [66]. Attitudes towards elephants may also affect the social acceptability of giving an electric shock to elephants using collars. This may vary significantly based on religious and cultural backgrounds and also depending on whether negative or positive interactions occur between humans and wild elephants [5,204]. Where negative perceptions are shown towards mitigation tools that have high efficacy, effort could be made to create awareness and change people’s attitudes towards such HEC mitigation tools. Hence, sociological surveys should be conducted to understand attitudes of various stakeholders at a preliminary stage to determine public opinion and acceptability of using AGDs on elephants in the future.

## 5. Conclusions

Elephants are endangered and play a significant role in the ecosystem and culture. Conflict between humans and elephants is one of the most important environmental issues in Asian elephant range countries. A variety of approaches are used to mitigate HEC, although most have not been very successful given they are not flexible or dynamic enough to be modified according to elephants’ behavioural and ecological needs. AGDs may overcome many of these issues, but require further development. AGDs may safely prevent elephant movement into human habitations and help humans and elephants coexist if elephants successfully learn to associate the non-aversive auditory stimulus with the aversive electric shock. Use of AGDs may be a more ethical choice than elephant removal. However, AGDs first require field-testing with captive elephants under controlled conditions to refine their design and optimise their efficacy and welfare impacts. Understanding public perceptions about AGDs is also important. AGDs will not be a ‘silver bullet’ for HEC, but they do overcome many of the limitations of current tools and may therefore become a powerful new management tool for reducing HEC in the future.

## Figures and Tables

**Table 1:** Summary of common human-elephant conflict (HEC) mitigation tools.

HEC Mitigation Tool	Function	Drawbacks and Non-Targeted Effects
1. Exclusion		
**Physical fences**		
i. Electric fences [68,69] ii. Non-electric fences e.g., trenches, rock walls and ditches [45,70,71]	Constructed to delineate a defined geographical area where managers can separate animals from people Can be effective where proper monitoring and sufficient funding for fence maintenance is available [72] In contrast to attempts at restricting elephants to small and permanently fenced areas, placing permanent electric fences around villages and temporary electric fences around agricultural lands, managed by local communities have been proven more effective [73,74]	Expensive to build and their location cannot be easily moved once constructed [75,76] Restrict access to critical food or habitat resources, disrupt movement and dispersal, and lead to isolation and fragmentation of populations for both elephants and non-target species [77,78,79] Problem may be solved locally but can be moved to another place [80] Elephants also learn to break electric fences [68,75] Trenches can be filled due to erosion and elephants kicking-in the sides [18,81]
**Bio fences**		
iii. Live fences- planting thorny plants like *Agave*, cacti, cane/rattan etc. [39,82]	Creating buffer zones using thorny plants that inflict mild pain and lacerations if ignored, surrounding commercial crop plants and home gardens to keep elephants away	Applicable only in very small scale [39] Require regular monitoring and maintenance [82] Thick-skinned elephants can push aside thorny shrubs or move through gaps created during planting [39,75]
iv. Planting non-preferred crops e.g., chilli, citrus, bitter gourd, okra, tea, coffee, aromatic medicinal plants etc. [18,82,83,84,85]	Planting non-preferred crops as a buffer zone or substituting attractive commercial crops with less attractive crops to keep elephants away May also provide an additional income to farmers	Some non-preferred plants (e.g., chilli and oranges) are known to be consumed by elephants at times [75] May not have a good market value and even if not consumed, damage may be caused by trampling them [39]
v. Beehive fences [71,86,87]	Beehive boxes fixed with ropes to fences are intended to repel elephants from crop fields as they fear the sting of the honeybee Bees’ honey may also provide an additional income to farmers	Using Asian honeybees *Apis cerana indica* may be ineffective for Asian elephants because Asian honeybees are not very aggressive or because they are active during daytime while elephants raid crops during the night [86] Bees may move away from boxes due to disturbance from humans, ants, or other animals [71]
**Other sensory deterrents**		
vi. Olfactory stimuli e.g., smoke and chilli bombs, chilli-grease fences [71,76,88,89,90] vii. Visual stimuli e.g., bonfires, flaming torches, lighting lamps, flashlights, light shining on compact disks hung on a string [45,89,91] viii. Acoustic stimuli e.g., shouting, fire crackers, carbide cannons, thunder flashes, drum beating, metal clanging, shot guns and playback calls [45,76,92,93]	Used as deterrents to chase or keep elephants away from human habitats and agricultural lands May be effective if used alternatively to avoid habituation	Cost effectiveness of chilli-grease fences in reducing crop raiding is uncertain because it is labour intensive to maintain as it require frequent reapplication and washes off during rain [71,75,76,90] Chilli bombs may have limited usage as wind direction cannot be controlled [39,71,89] Elephants have suffered burn injuries due to flame torches being thrown at them, heightening risk of mortality [94,95] Elephants habituate to these methods and sometimes even act aggressively in response to them [73,89,94] Targets only small, localised areas (e.g., small village, paddy field etc.)
ix. Elephant drives [18,27,96,97]	Elephants are pushed out of human habitats and into protected areas using people, vehicles, aircrafts, or trained elephants	Large-scale elephant drives are very costly, time consuming, require considerable human resources and mainly drive away family herds but not the problem-causing lone male elephants [27,39] Poses a risk to the people involved in moving elephants Driven elephants become concentrated into small areas with insufficient resources and then suffer starvation or escape or leave these areas, repeating the cycle of HEC [27] Causes severe stress to elephants [98]
2. Removal of individual problem elephants		
i. Translocation	Targeted problem elephants are tranquilised and transported away from their capture site to protected areas [99,100,101], wild elephant holding grounds [102] or alternatively captured and tamed [103,104]	Expensive operation [35,73,105] regardless of whether translocated to other wilderness areas or into captivity Translocated elephants typically do not stay in the areas where they are released, but instead return to their place of capture or disperse and settle in new areas and create new conflicts merely shifting the conflict from one place to another [2,100,101,106] Elephant holding grounds are expensive to build and maintain, hormonal and reproductive control is required, and the facilities can only house a relatively small number of animals which may quickly reach capacity [107,108] Difficulty in the taming process of wild and mature elephants which may also result in injury, trauma and subsequent death of the animal [108,109] Increasing cost of maintenance of the high number of captured problem elephants in captivity [110]
ii. Killing of problem elephants	Identified problem individuals known to cause frequent HEC incidents may be killed, aiming to eliminate the problem from the area [75]	Degrade the genetic diversity of a population and impractical depending on the scale of HEC [108] Using lethal methods to resolve HEC is controversial and considered ethically unacceptable in most contexts [108,111]
3. Early warning systems		
i. Traditional early warning systems e.g., watch huts and iron watch towers [76,82,112,113]	Places from where people can monitor elephants and alert farmers and villages to scare and chase elephants away and prevent crop damage	Labour intensive Loss of sleep at night may affect personal health and social wellbeing of farmers [52,114]
ii. Modern remote sensing methods e.g., Global Positioning System (GPS) collars [115], infrasonic call detectors [116], geophones [117] trip wire systems [71], drones and infrared triggered cameras [118]	Monitoring elephant movement remotely using emerging technologies to warn authorities and villagers via automatically triggered sirens or phone messages when elephants are in close proximity to human habitats to prevent accidental encounters with elephants.	Limited battery life of GPS collars, high risk and cost of collaring process [119] Risk of damage to devices by elephants and people [71] Development and installation of technology requiring large amount of financial resources [118] Requires uninterrupted satellite and mobile network communication in remote and heterogenous landscapes for real-time monitoring of elephants
4. Human centric methods		
i. Providing financial relief e.g., compensation and insurance schemes [48,49,50,120]	Financial support as compensation or through insurance schemes to provide immediate relief from elephant impacts [121]	Impact assessments are subjective and difficult [122] Process of reporting incidents and claiming compensation may be complicated and time consuming [50,120,122,123] Amount of funds available are inadequate, are subject to fraudulent claims and corruption [39,123,124] Depending on the extent of HEC, assessment of damage could be quite labour intensive [122]
ii. Creating awareness and capacity building [29,118,125,126,127]	Educating local people about the importance of elephants, and how to prevent or reduce encounters with elephants or protect themselves to improve people’s perception towards elephants Training stakeholders especially wildlife officers and local communities to handle HEC situations and empowering local communities by providing resources for alternative income generation to help change people’s attitudes	Requires post-monitoring to ensure that human attitudes, behaviours and practices have actually changed given that information can easily be misinterpreted or ignored [128]
5. Habitat management		
i. Improving connectivity between habitats [129,130]	Creating or securing forested paths between elephant habitats with minimum human interference to reduce HEC incidents [110]	Need for legal protection to ensure these corridors are secured and regular monitoring of corridors [129] Financial commitment for monitoring and maintenance of these corridors [130]
ii. Improving habitat quality inside protected areas [55,82,131,132]	Increasing carrying capacity inside protected areas by creating and maintaining salt licks, managing water sources, planting fodder species, maintaining grassland areas and removing invasive species etc. to attract elephants, thereby managing their distribution	Increased densities of elephants resulting from improved habitat quality may not be sustainable due to overutilisation [133] Selectively bred cultivated crops are also known to be more palatable and attractive for elephants regardless of the availability of other food [134] Require regular monitoring and maintenance of salt licks and water holes as well as plants until they are established [81]

**Table 2:** Aversive conditioning tools used in attempts to mitigate conflict between humans and Asian elephants.

Tool	Warning Signal	Aversive Stimuli
Electric fences	Visual	Electric shock, mild pain
Trenches, canals, ditches etc.	Visual	Injury and immobility
Thorny plants	Visual	Mild pain, pricks, lacerations
Non-preferred crops	Visual and olfactory	Unpleasant taste
Bee fences	Auditory visual and olfactory	Painful bee sting
Smoke, chilli bombs	Visual and olfactory	Uncomfortable olfactory stimulus
Bonfires, flashlights, flaming torches etc.	Visual	Uncomfortable visual stimulus
Shouting, thunder flashes, firecrackers, carbide cannons, playback of calls (e.g., carnivore growls) etc.	Auditory	Fear- inducing uncomfortable auditory stimulus

**Table 3:** Summary of studies conducted with wild species using Aversive Geofencing Devices (AGDs).

Study	Species (Captive/Wild)	No of Shock-Collared Animals	Aim	Delivery of Stimuli	Outcome
Andelt et al. (1999) [157]	Coyote (captive)	5	Prevent attacks on livestock	Manual: Shock delivered as the coyote actively pursued a lamb and was about 2–5 m from the lamb.	Shock collars were successful in preventing attacks during all attempts (n = 13) by coyotes to attack lambs. The probability of attacks on lambs decreased and the coyotes avoided, retreated, and even showed submissive behaviours towards lambs. No attacks were attempted by coyotes during the last four months of the study showing sustained effects of aversive conditioning.
2. Appleby, (2015) [158]	Dingo (wild)	4	Mitigating human-wildlife conflict	Manual?	During a series of trials conducted with shock collars, two dingoes responded to the shock by immediately halting the problem behaviour. The third animal became hesitant to approach a target after receiving two shocks over a few days. The fourth animal tested consistently fled after receiving a shock no matter what target behaviour was involved.
3. Cooper et al. (2005) [159]	Island fox (wild)	~68/year	Prevent attacks on nests of an endangered species	Automatic: An antenna transmitting a signal, activated the shock collars if the animal approached within ~1–2 m of the transmitting antenna wire placed around a nest tree.	Study showed that shock collars have the potential to manage predators from approaching nests. The nests protected by antennae transmitting signals were more successful (64%) than those that were not protected (31%). However, high success rate of the protected nests was also due to multiple aspects that were involved during the study and not only due to fox deterrence.
4. Gehring et al. (2006) [160]	Gray wolf (wild)	5	Area avoidance to prevent livestock depredation	Automatic: Collars activated automatically when detected 30–70 m from the transmitter.	A 14-day shock period was successful in reducing the frequency of approaches by wolves to baited sites by 50%. The study was then successful in preventing all pack members in five shock-collared wolf packs to avoid shock sites for more than 60 days after being exposed to shocks over a 40-day period.
5. Hawley et al. (2009) [161]	Gray wolf (wild)	5	Area avoidance to prevent livestock depredation	Automatic: Transmitters maintaining a shock zone with a 30 m radius, activated collars when the animal entered the shock zone.	Shock collared wolves spent less time and made fewer visits to baited sites compared to control animals during shocking period. But it is not clear if wolves were successfully conditioned because only a slight reduction in visitation was observed during post-shocking period with the shock collared wolves.
6. Hawley et al. (2013) [162]	Gray wolf (captive)	16 *	Improve shock collar design	Manual: Activation using a hand-held device.	This study tested and improved shock collar designs for safety and efficacy to eliminate neck damage and was able to extend the battery life of the collar up to 80 days while effectively delivering a shock.
7. Nolte et al. (2003) [163]	Black-tailed deer (captive)	6	Area avoidance to reduce food competition with livestock	Automatic: A sound followed by an electric shock was emitted from the collar when the animal approached a plot with a signal emitting wire buried beneath the ground around its perimeter.	Deer successfully learnt to avoid areas associated with the shock. However, avoidance of previously shocked areas (plots) stopped sometime after shock collars were deactivated.
8. Rossler et al. (2012) [164]	Gray wolf (wild)	10	Area avoidance to prevent livestock depredation	Automatic: Collars activated when wolves were within a 70 m radius around the bait site.	Visitation and time spent in shock zones by shock-collared wolves were less compared to control wolves during the 40-day shock period and the 40-day post-shock period. During this study, shock collars were able to condition wolves to avoid specific sites long after the shocking period and reduce visitation by other pack members not wearing shock collars indicating social facilitation.
9. Schultz et al. (2005) [165]	Gray wolf (wild)	2	Area avoidance to prevent livestock depredation	Manual and automatic: Wolf was shocked using a hand-held unit every time her location indicated travel within 300 m of the cattle pasture during a preliminary study. A proximity-based sensor was then used to automatically emit a beep and a shock when the animal came within 400 m of the device.	Preliminary study showed that manually activated shock collar could keep a wolf away from a farm; however, it did not have a long-term effect on the wolf’s behaviour. A wolf that was receiving a beep before the shock automatically and had learnt to avoid the farm successfully, later reacted to the sound warning alone and moved about 800 m away from the beeper within 7 min avoiding the shock. In contrast two other wolves who were not wearing shock collars either did not move at all or moved towards the target in response to the beeper.
10. Shivik and Martin, (2000) [166]	Gray wolf (wild#)	3	Prevent attacks on livestock	Automatic: Shock collar on the wolf activated if it approached within ~1 m of the calf wearing an electronic device emitting signals.	Electric shock repelled wolves from calves and wolves did not attempt an attack after the first conditioning experience. The study showed that giving the shock at ~1 m helped wolves to recognise their undesirable behaviour and maintained distance from calves.
11. Shivik et al. (2002) [167]	Gray wolf (wild#)	5	Prevent attacks on livestock	Automatic: Shock collar on the wolf activated if it approached within ~1 m of the calf wearing an electronic device emitting signals.	Unsuccessful in conditioning wolves not to attack livestock due to various logistical and behavioural reasons.
12. Shivik et al. (2003) [168]	Gray wolf (captive)	10?	Area avoidance to prevent livestock depredation	Automatic: Signal emitting wires buried beneath the area of the food source activated the collar if a wolf approached within 2 m of the food source.	Study was not very successful in conditioning captive wolves with training collars due to logistical and behavioural variability.

* Four or six animals used in each of the five trials. Same animals may have been re-used in some trials. # Wild, but animals were temporarily held in captivity. ? indicates uncertainty.

## Data Availability

Not applicable.

## Appendix A

**Table A1:** Key findings of some research conducted on virtual fencing with Aversive Geofencing Devices (AGDs) on livestock published between 2017–2022.

	Study	Country	Summary
1	Aaser et al. (2022) [205]	Denmark	AGDs were successful in keeping the cattle within the virtual fences with no acute welfare impacts. However, there were individual variations between cows in their responses and were also influenced by stimuli received by other herd members.
2	Boyd et al. (2022) [62]	USA	This study focussed on excluding cattle from recently burned areas and AGDs were quite effective in limiting the use of burned areas by cattle.
3	Brunberg et al. (2017) [206]	Norway	The prototype device used was not very successful in keeping the sheep within the restricted zones and animal welfare may not be assured with this system.
4	Campbell et al. (2017) [154]	Australia	Cattle were able to associate the audio cue with the aversive stimuli from the AGDs and avoid moving virtual fences, thus animals did not associate the aversive stimuli with the location but responded to the audio cue from the collar.
5	Campbell et al. (2018) [155]	Australia	AGDs were able to successfully exclude most cattle from accessing a feed attractant but the rate of learning highly differed between individuals.
6	Campbell et al. (2019a) [207]	Australia	AGDs were successful in temporarily excluding a group of cattle from a riparian zone and animals re-entered the previously excluded area after fence deactivation.
7	Campbell et al. (2019b) [193]	Australia	AGDs were effective in containing cattle within a virtual fenced area without much impact on physiological stress levels or behavioural time budgets and showed no difference compared with those animals within a physical electric fence.
8	Campbell et al. (2020) [61]	Australia	AGDs were able to successfully exclude a group of cattle from an environmentally sensitive area across a period of 44 days, with the feed available in the protected zone doubled by the end of the experiment.
9	Campbell et al. (2021) [208]	Australia	Preliminary trials conducted on cattle and sheep demonstrated the potential to use AGDs for herding animals, however, further experimentation with updated versions of the device is required.
10	Colusso et al. (2020) [209]	Australia	Cows were trained to learn and respond to AGDs as individuals and in groups. When those trained in groups were tested individually, they were more likely to interact with virtual fences than those initially trained individually and then later tested in groups. This study demonstrated that those trained in groups relied on the responses of their conspecifics and for accurate learning of virtual fences, it is important that individual animals directly receive stimuli.
11	Colusso et al. (2021a) [210]	Australia	Experiments conducted with AGDs to evaluate the impact of feed restriction showed that the restriction of food may impact the exclusion of cows from a feed attractant, but later they quickly learnt to avoid receiving the electrical stimuli and stayed within the restricted zone.
12	Colusso et al. (2021b) [211]	Australia	AGDs were successful in excluding cows from fresh pasture even when they were only provided with post-grazing residuals. However, there were individual variations in the number of stimuli received by animals and time spent in the exclusion zone.
13	Kearton et al. (2019) [200]	Australia	Experiment was conducted to understand the stress responses of sheep to AGDs compared to other commonly encountered stimuli such as a barking dog and restraint procedures. Results showed that electric stimuli on sheep had no significant effect on physiological stress levels and showed aversive behavioural responses that were less aversive compared to commonly practiced restraining procedures.
14	Kearton et al. (2020) [172]	Australia	Predictability and controllability of the aversive stimuli from AGDs minimises both physiological and behavioural stress responses during aversive conditioning.
15	Kearton et al. (2022) [212]	Australia	Maternal demonstrators exposed to virtual fences with AGDs may contribute to the learning of virtual fences by lambs. However, this study protocol was limited by several aspects and therefore, further exploration of this is recommended.
16	Keshavarzi et al. (2020) [178]	Australia	This study showed that cattle learned to avoid virtual fences through social facilitation where animals stayed within a restricted zone based on the response of conspecifics.
17	Langworthy et al. (2021) [213]	Australia	Virtual fencing using AGDs were 99% successful in containing a herd of dairy cows within a restricted zone compared to the physical electric fences.
18	Lomax et al. (2019) [63]	Australia	AGDs were successful in keeping cows within a designated area 99% of the time, however learning rate of individual animals varied.
19	Marini et al. (2018a) [214]	Australia	Over a period of 3 days, after an average of 8 interactions, sheep learned to associate the auditory cue with the aversive stimuli. After the collar was removed, the sheep moved into the exclusion zone after 30 min.
20	Marini et al. (2018b) [153]	Australia	Mean of three trials were required for the sheep to learn to associate the auditory cue with the electrical stimuli. After that 52% of the sheep avoided receiving the electric shock after hearing the auditory signal.
21	Marini et al. (2019) [215]	Australia	The group of sheep that received both an auditory cue followed by electrical stimuli were able to predict the receipt of electrical stimuli and thus showed more favourable responses to the fence compared to the group that only received an electrical cue. Animal’s temperament showed no relationship on its learning ability.
22	Marini et al. (2020) [177]	Australia	The experiment with sheep showed that collaring 66% of a flock was enough to contain the entire flock within the exclusion zone indicating that sheep learn through social facilitation. However, collaring 33% of the flock did not prevent the flock from entering the exclusion zone.
23	Marini et al. (2022) [216]	Australia	Study showed that virtual fencing is as effective as electric fencing and virtual fenced sheep did not differ in their normal grazing behaviour.
24	McSweeney et al. (2020) [217]	Ireland	When visual boundaries were removed, cows made more boundary challenges. Also, cows grazed less in inclusion zone implying they were stressed.
25	Muminov et al. (2019) [218]	Korea	Goats responded positively to both electric shock and warning sounds. Also, the designed collar was effective at automatically classifying main behaviour categories.
26	Ranches et al. (2021) [219]	USA	Cows showed increased distressed behaviours when first fitted with the collars. However, they quickly adapted to the AGD. Cows also learned to avoid the exclusion zone when fitted with an AGD. Upon removing the AGD cows resumed normal behaviours.
27	Verdon et al. (2020) [181]	Australia	Study shows that cows that have had prior experience with electric fences learn the virtual fence techniques much faster.
28	Verdon and Rawnsley, (2020) [220]	Australia	Older heifers (22 months) learn to avoid the electrical stimuli quicker than younger animals (12 months). When the younger animals were re-trained at 22 months, they did not show a significant difference compared to the original 22-month animals. This showed that prior learning at a young age does not have an effect in avoiding the electrical stimuli later in life.
29	Verdon et al. (2021a) [221]	Australia	The study comprised of four groups of cattle grazing in adjacent paddocks, where two control groups were contained within physical electric fences and the other two with AGDs. AGDs successfully contained one group of animals, but the second group frequently encroached the exclusion zone. Study suggested that when animals have visual contact of other conspecifics in adjacent paddocks, the efficacy of AGDs can be reduced.
30	Verdon et al. (2021b) [222]	Australia	Milk production, live weight and standing and lying behaviour budgets did not differ between electric and virtual fence cattle groups. There was no significant welfare or behaviour effects immediately following implementation of AGDs (days 1–3). However, there was an increase in milk cortisol and changes in behavioural time budgets later (after day 4) with the virtual fence group. Therefore, a longer study period is required to determine the welfare impacts of AGDs on lactating dairy cattle
